# Fornix deep brain stimulation enhances acetylcholine levels in the hippocampus

**DOI:** 10.1007/s00429-015-1144-2

**Published:** 2015-11-24

**Authors:** Sarah Hescham, Ali Jahanshahi, Judith V. Schweimer, Stephen N. Mitchell, Guy Carter, Arjan Blokland, Trevor Sharp, Yasin Temel

**Affiliations:** 1 Department of Neuroscience, Maastricht University, P.O. Box 616, 6200 MD Maastricht, The Netherlands; 2 Department of Neuropsychology and Psychopharmacology, Maastricht University, P.O. Box 616, 6200 MD Maastricht, The Netherlands; 3 Department of Neurosurgery, Maastricht University Medical Centre, PO Box 5800, 6202 AZ Maastricht, The Netherlands; 4 Eli Lilly and Company Ltd., Windlesham, GU20 6PH UK; 5 University Department of Pharmacology, University of Oxford, Oxford, OX1 3QT UK; 6 European Graduate School of Neuroscience (Euron), Maastricht University, Maastricht, The Netherlands

**Keywords:** Deep brain stimulation, Memory, Fornix, Hippocampus, Acetylcholine

## Abstract

Deep brain stimulation (DBS) of the fornix has gained interest as a potential therapy for advanced treatment-resistant dementia, yet the mechanism of action remains widely unknown. Previously, we have reported beneficial memory effects of fornix DBS in a scopolamine-induced rat model of dementia, which is dependent on various brain structures including hippocampus. To elucidate mechanisms of action of fornix DBS with regard to memory restoration, we performed c-Fos immunohistochemistry in the hippocampus. We found that fornix DBS induced a selective activation of cells in the CA1 and CA3 subfields of the dorsal hippocampus. In addition, hippocampal neurotransmitter levels were measured using microdialysis before, during and after 60 min of fornix DBS in a next experiment. We observed a substantial increase in the levels of extracellular hippocampal acetylcholine, which peaked 20 min after stimulus onset. Interestingly, hippocampal glutamate levels did not change compared to baseline. Therefore, our findings provide first experimental evidence that fornix DBS activates the hippocampus and induces the release of acetylcholine in this region.

## Introduction

Dementia is a major threat to human population. The World Health Organization estimates that 35.6 million people suffer from dementia, a number that is anticipated to triple by 2050 (Batsch and Mittelman 2012). The most common form of dementia is Alzheimer’s disease (AD), accounting for 60–80 % of all cases. So far there are no known treatments available that cure or delay the progression of AD. Current pharmacological treatments are not effective for every patient and only alleviate symptoms temporarily (Rosini et al. 2008; Thies and Bleiler 2011).

Recently, the application of DBS to reduce or delay the progression of memory loss in AD has shown to be effective. In particular, the convergence of the latest observations in humans and rats pinpoints that DBS of the fornix can restore memory loss (Hamani et al. 2008; Hescham et al. 2013; Laxton et al. 2010). In our own experiments (Hescham et al. 2013), fornix DBS reduced memory deficits induced by the muscarinic receptor antagonist scopolamine in a rat model. In this scopolamine-induced rat model of memory impairment, various brain structures including the hippocampus are affected (Hescham et al. 2014). The mechanism of action of fornix DBS is not well understood but the latter suggests an involvement of the hippocampus and acetylcholine.

In the present study, we therefore investigated the effects of fornix DBS on the hippocampus with a dual approach. First, we evaluated c-Fos expression in the dorsal hippocampus. The immediate early gene c-Fos is an indirect marker of neuronal activation and can be even used to map long-term activation (Budzikowski et al. 1998; Jahanshahi et al. 2013). Second, we investigated the effect of fornix DBS on levels of hippocampal acetylcholine as well as glutamate; neurotransmitters which are widely acknowledged as important for memory functions (Klinkenberg et al. 2011; Micheau and Marighetto 2011).

## Materials and methods

### Experimental design

This study consists of two experiments:

*Experiment 1* Male Sprague–Dawley rats (Charles River, Sulzfeld, Germany) were either assigned to the fornix DBS (*n* = 5) or sham (*n* = 5) group. Electrode implantations were performed as described previously (Hescham et al. 2013). DBS electrodes were implanted at the site of the fornix (coordinates from bregma according to the rat brain atlas of Paxinos and Watson (Paxinos and Watson 1998): AP: −1.8 mm; ML: 1.3 mm; DV: −8.0 mm). It was found that fornix DBS reduced memory deficits induced by the muscarinic receptor antagonist scopolamine in a hippocampal-dependent rat model as reported elsewhere (Hescham et al. 2013). In these same animals, 1 week later (when scopolamine is no longer present), rats were stimulated at 100 Hz, 100 µA and 100 µs pulse width for 1 h and allowed to rest for 1 h. Sham rats were only attached to cables, but not stimulated. Subsequently, animals were transcardially perfused, first with Tyrode’s solution and then Somogyi fixative (Somogyi and Takagi 1982). Animal procedures in experiment 1 were approved and carried out in accordance to the Animal Experiments and Ethics Committee of Maastricht University.

*Experiment 2* To establish whether fornix DBS affects the neurochemistry in the hippocampus, microdialysis experiments were carried out in male Sprague–Dawley rats (Harlan, Bicester, U.K., fornix DBS *n* = 13 and sham *n* = 6) under urethane anesthesia. Animal procedures in experiment 2 were carried out in accordance with the UK Animals (Scientific Procedures) Act 1986 and associated Home Office guidelines, and with local ethical approval.

### Immunohistochemistry

Following transcardial perfusion in experiment 1, brains were collected and cut into 30 µm slices on a vibratome (Leica^®^, Wetzlar, Germany). For immunohistochemistry, sections were incubated overnight with polyclonal rabbit anti-c-Fos primary antibody (1:2000; K-25, Santa Cruz Biotechnology Inc, Santa Cruz, USA) followed by biotinylated donkey anti-rabbit secondary antibody (1:800; Jackson Immunoresearch Laboratories Inc., Westgrove, USA) and avidin–biotin peroxidase complex (1:800, Elite ABC-kit, Vectastain^®^, Burlingame, CA, USA). The staining was visualized by 3,3′-Diaminobenzidine (DAB) combined with NiCl_2_ intensification.

Images were taken at two similar bregma levels using a U-CMAD-2 digital camera connected to an Olympus AX70 brightfield microscope (analySIS; Imaging System, Münster, Germany), and evaluated with ImageJ (Image J software version 1.38x; NIH, Bethesda, USA). C-Fos-positive cells in the medial prefrontal cortex and hippocampus were counted manually by an observer blind to treatment.

### In-vivo microdialysis

For experiment 2, rats were anesthetized with 1.3-1.5 g/kg urethane (ethyl carbamate, Sigma) and mounted in a stereotaxic frame. DBS electrodes were implanted at the site of the fornix, and a single cannula microdialysis probe (CMA11, tip length 2 mm, CMA Microdialysis, Kista, Sweden) was implanted into the hippocampus (coordinates from bregma: AP: −4.8 mm; ML: 3 mm; DV: −4.2 mm). Microdialysis probes were perfused with artificial cerebrospinal fluid (141 mM NaCl, 5 mM KCl, 0.8 mM MgCl_2_, 1.5 mM CaCl_2_) at a flow rate of 1.5 µl/min (CMA/100, Carnegie Medicine) for 2 h before dialysate collection started. Dialysate samples were collected every 20 min, DBS stimulation time (100 Hz, 100 µA and 100 µs) was 1 h. In total, 10 samples were collected (4 baseline, 3 during stimulation and 3 after stimulation). Samples were immediately frozen on dry ice and later analyzed with liquid chromatography/mass spectrometry.

### Liquid chromatography/mass spectrometry

All microdialysis samples were analyzed at Eli Lilly and Company Ltd., Windlesham, U.K. The high-performance liquid chromatography (HPLC) system consisted of a PAL HTC-xt autosampler (CTC Analytics AG Zwingen, Switzerland), a pair of Shimadzu LC-20AD_XR_ pumps with a Shimadzu CBM-20A controller and Shimadzu CTO-20AD column oven (Shimadzu Ltd., Milton Keynes, UK). Injection volume was 10 µl. The chromatographic retention was obtained using an XBridge BEH Amide column (75 × 2.1 mm, i.d. 2.5 µm; Waters Ltd, Elstree, U.K.) at 35 °C. The gradient elution was carried out using acetonitrile and 2 mM ammonium formate, 95:5 and 5:95 (the pH of 3.0 was adjusted with formic acid). Detection was carried out by an AB Sciex Triple Quad API5500 mass spectrometer MS (AB Sciex UK Ltd, Warrington, U.K.), which operated in TurboIonSpray^®^ mode using a Turbo V™ source and SRM analysis.

Acetylcholine was detected by monitoring the *m/z* 146.1 → 87.0 transition and its D4 analog internal standard at *m/z* 150.1 → 91.0 (dwell time: 50 ms, collision energy: 20 V, collision cell exit potential: 4.2 V). Glutamic acid was detected by monitoring the *m/z* 148.0 → 84.0 transition and its D5 analog internal standard at *m/z* 153.0 → 88.0 (dwell time: 50 ms, collision energy: 23 V, collision cell exit potential: 10 V). Samples were prepared by 1:10 dilution in internal standard and a single acetylcholine calibration curve between 0.10 and 20.0 nM and glutamic acid curve between 50 and 2000 nM was run at the end of each batch of samples for quantification.

### Verification of DBS electrodes and microdialysis probes

Sections containing the electrode trajectories from all animals were collected and mounted on gelatin-coated glass slides. A standard haematoxylin–eosin staining was carried out before sections were photographed under bright field microscopy.

### Statistical analysis

For experiment 1, the number of Fos-positive cells was expressed as percentage of change when compared to sham (i.e., the average of Fos-positive cells in sham was chosen as reference value and defined as 100 %). Raw values as well as percentage scores were normally distributed as assessed by the Shapiro–Wilk’s test. For better comparison between the groups, however, we chose to present the percentage scores. An independent-samples *t* test was employed and *p* values < 0.05 were considered significant. Microdialysis data of experiment 2 were represented as percentage of the mean of 4 baseline samples prior to DBS. The effect of fornix DBS on extracellular acetylcholine and glutamate levels over time was analyzed by repeated-measures ANOVA followed by Fisher’s least significant difference (LSD) post hoc test.

## Results

### Verification of DBS electrodes and microdialysis probes

The location of all DBS electrodes was verified within the fornix, and microdialysis probes were all correctly placed in the dorsal hippocampus. For the current stimulation settings used, we found no evidence for histological damage as observed by a standard haematoxylin–eosin staining.

### Immunohistochemistry

**Fig. 1:**
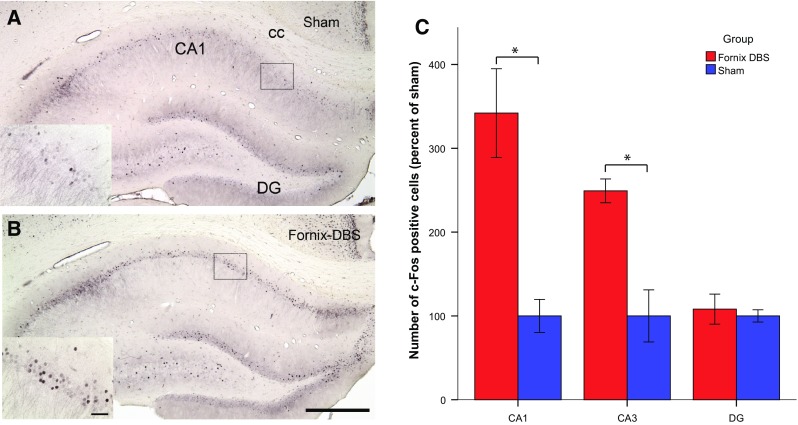
Representative low-power photomicrographs (*scale bar* = 500 µm) of coronal brain sections stained for c-Fos (K-25) showing the hippocampus of sham (**a**) and fornix stimulated animals (**b**). The high-power photomicrograph insets in the lower left corner show the CA1 subregion of the hippocampus (*scale bar* = 50 µm). **c** Comparisons were made as percentage increase/decrease with regard to sham. Fornix DBS rats show increased c-Fos (K-25) expression in the CA1 and CA3 subregion of the hippocampus when compared to sham. There was no statistical difference between sham and DBS groups in the dentate gyrus. *p < 0.05, independent-samples *t* test for fornix DBS vs. sham rats. Data represent mean ± SEM. *DG* dentate gyrus, *cc* corpus callosum

We found increased c-Fos expression in the CA1 subregion for fornix stimulated animals when compared to sham [*t*(8) = 4.285; *p* < 0.01]. In addition, increased levels of c-Fos cells in the CA3 subregion were observed in fornix DBS rats [*t*(8) = 4.363; p < 0.01]. There was no statistical difference between levels of c-Fos expression in the dentate gyrus of fornix stimulated and sham rats [*t*(7) = 0.455; n.s.; Fig. 1].

### In-vivo microdialysis

**Fig. 2:**
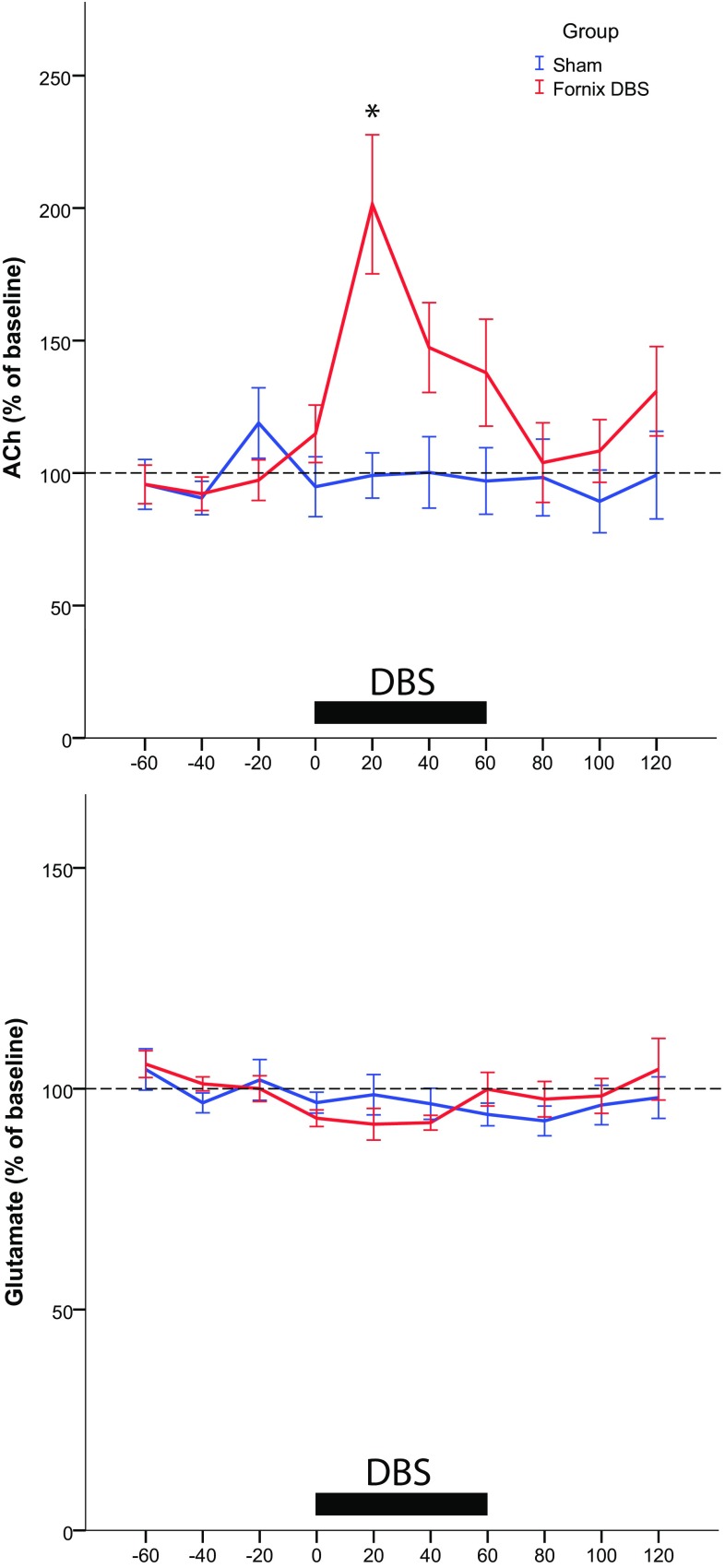
Microdialysate acetylcholine (ACh, **a**) and glutamate (**b**) levels of the dorsal hippocampus in anesthetized fornix DBS (*n* = 13) and sham rats (*n* = 6). *Horizontal bar* stimulation period. ACh levels were significantly elevated in the fornix DBS group after 20 min of stimulation. No difference in glutamate levels was detected between fornix DBS and sham. Data points are mean ± SEM expressed as  % of baseline. Mean raw values for baseline ACh were 0.324 nM ± 0.023 and for glutamate 3.05 µM ± 0.24. *p < 0.05, repeated-measures ANOVA for fornix DBS vs. sham rats

Fornix DBS for 60 min caused a significant increase in hippocampal acetylcholine levels in comparison to non-stimulated controls [repeated-measures ANOVA: *F*(1,17) = 6,608; *p* < 0.03]. This effect was evident in the first 20 min of fornix DBS as shown by the LSD post hoc test (*p* < 0.03; Fig. 2a). Acetylcholine levels in other time points during stimulation as well as after stimulation did not differ between the groups.

There was no statistical difference for hippocampal glutamate levels between fornix DBS and sham [repeated-measures ANOVA: *F*(1,16) = 0.038; n.s.; Fig. 2b].

## Discussion

According to several studies, fornix DBS in AD patients leads to a decreased rate of cognitive decline (Fontaine et al. 2013; Laxton et al. 2010); yet, a potential mechanism of action has not been found. In the present study, we found that beneficial memory effects of fornix DBS might be linked to an enhanced neural activity in the CA1 and CA3 subfield, suggesting increased activity of certain neurons in this region. The CA1 and CA3 subregions of the hippocampus are important for spatial memory (Farovik et al. 2010; Lee et al. 2005). In particular, the CA3 subregion plays a role in encoding and retrieval of spatial location sequences and the CA1 contributes to memory encoding by binding cues to their temporal context, which in turn also enables retrieval of location sequences (Farovik et al. 2010). Interestingly, a substantial neuronal loss in the CA1 is observed in post-mortem AD brains (West et al. 2004). If fornix DBS is able to increase neural activity in the CA1 subfield, then it may compensate for reduced neuronal integrity of this region in AD.

In the etiology of AD, evidence exists for both cholinergic and glutamatergic involvement (Francis 2005). In the present study, microdialysis data revealed that hippocampal glutamate levels were not affected by fornix DBS. A clear connection between glutamate and memory-related processes is provided by the association of ionotropic glutamate receptors in the induction of long-term potentiation. Although memory can be disrupted by blockade of these receptors (e.g., *N*-methyl-d-aspartate, NMDA) (Morris et al. 1986), the enhancement of the glutamatergic signal may also have adverse effects on memory, because high levels of glutamate are neurotoxic (Lau and Tymianski 2010). Our results suggest that fornix DBS does not influence glutamate-dependent long-term potentiation, but also does not induce excitotoxicity.

Contrary to this, fornix DBS did have an effect on extracellular hippocampal acetylcholine and it might be possible that increased c-Fos expression in the hippocampus was mediated by enhanced acetylcholine levels (Narimatsu et al. 2009). In an earlier study, we have shown that fornix DBS was able to restore scopolamine-induced memory deficits when stimulating the fornix for 5 min during the acquisition and retention trial of the object location task, a test of hippocampal-dependent memory (Hescham et al. 2013). Potentially, the increased hippocampal acetylcholine measured via microdialysis after 20 min facilitated the enhanced memory performance. However, these results should be interpreted with caution, since we did not include any memory tests in the present study. The effect of DBS (20 min) on acetylcholine release seemed to decline with continued stimulation. This transient effect can be explained by exhaustion of the acetylcholine pools that may take place after an enhanced release for more than 20 min. Thus, chronic DBS might eventually be associated with acetylcholine depletion. In this case, either intermittent or closed-loop stimulation (Rosin et al. 2011) when applying chronic DBS of the fornix might be necessary to maintain beneficial effects on memory functions.
